# Opportunistic Recharge Enhancement in Arid and Semi‐Arid Regions

**DOI:** 10.1111/gwat.70070

**Published:** 2026-04-14

**Authors:** Neha Gupta, Ryan Lima, Temuulen Tsagaan Sankey, Caelum Mroczek, Katharine Jacobs, Rayni Lewis, Fern Bromley, Tianfang Xu, Holly Richter, Patrick Broxton, Yoganand Korgaonkar, Travis Zalesky, James Famiglietti, Abraham Springer

**Affiliations:** ^1^ School of Earth and Sustainability Northern Arizona University Flagstaff AZ; ^2^ Arizona Institute for Resilience, Center for Climate Adaptation Science and Solutions University of Arizona Tucson AZ; ^3^ School of Natural Resources and the Environment University of Arizona Tucson AZ; ^4^ School of Sustainable Engineering and the Built Environment Arizona State University Tempe AZ; ^5^ Resilient Rivers LLC Hereford AZ; ^6^ School of Geography, Development, and the Environment University of Arizona Tucson AZ; ^7^ School of Sustainability Arizona State University Tempe AZ

## Abstract

Groundwater supplies are stressed by climatic trends, increasing withdrawals, and multiple competing uses, especially in arid and semi‐arid environments. We propose opportunistic recharge enhancement (ORE) as a cross‐disciplinary, scalable framework to augment groundwater supplies by strategically integrating recharge co‐benefits into existing land and water management practices such as forest thinning and stormwater management. These opportunities would allow for enhanced recharge across diverse landscapes, potentially increasing water availability for both human uses and natural systems. A key incentive for ORE is that, while there may be some increases in cost in some cases, the benefits and opportunities for funding may be significantly expanded. Using Arizona, USA, a region experiencing acute groundwater stresses, as an example, we illustrate how ORE can offer a complementary pathway to groundwater resilience with potential to extend to semi‐arid and arid areas globally.

## Graphical abstract

Groundwater supplies are increasingly stressed by climatic trends and multiple competing uses, especially in dryland environments. We propose opportunistic recharge enhancement (ORE) as a cross‐disciplinary, scalable framework to augment groundwater supplies by strategically integrating recharge co‐benefits into existing land and water management practices to support human uses and natural systems.

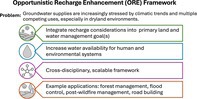

## Introduction

Groundwater is a critical resource, with depletion issues getting increased attention globally (Konikow and Kendy 2005; Famiglietti 2014) and nationally within the United States (PCAST 2024; Alley et al. 2025). Water managers in arid regions are heavily reliant upon groundwater supplies as vital buffers against variable surface water supplies for sustaining both ecological processes and human needs (Scanlon et al. 2006; Aeschbach‐Hertig and Gleeson 2012; Rohde et al. 2024). However, this resource is increasingly stressed by agricultural irrigation, urbanization, and the compounding effects of climate change, including altered precipitation patterns, increased evaporation, and extended droughts, resulting in increased demand for groundwater (Taylor et al. 2013). Global drylands are also experiencing an increasing discrepancy between predicted and observed hydroclimate, with lower moisture availability than anticipated (Reager et al. 2016; Simpson et al. 2024). These increasing pressures on water availability and demand further complicate the management of inherently complex groundwater systems.

Managed aquifer recharge (MAR) methods have been adopted as groundwater management strategies to increase aquifer recharge at rates exceeding natural processes; they can help offset groundwater declines due to pumping and/or to support groundwater‐dependent ecosystems. While there are many examples of MAR projects in arid regions globally (e.g., Fathi et al. 2021), there has been little consideration of recharge as a potential co‐benefit in other types of land and water management efforts (e.g., forest thinning, fire risk reduction, flood management, etc.).

Here, we propose an opportunistic recharge enhancement (ORE) framework that bridges traditional MAR approaches and non‐groundwater specific resource management actions by strategically integrating recharge co‐benefits into the latter practices. Our approach is based on our observations that recharge‐related opportunities are commonly missed in the context of land development, and in many cases, land use and land management decisions are damaging existing natural recharge capacity. Applying the ORE approach does not necessarily imply any inherent increase in the cost of management actions; rather, in specific circumstances significant benefits may be accrued without substantial additional investments and in others the incremental cost can be minimal relative to overall project costs. Applying cross‐disciplinary insights from ecohydrology and ecohydrogeology (Cantonati et al. 2020), we illustrate this concept with examples from Arizona, USA.

Groundwater provides more than 40% of Arizona's total annual water supply. Arizona is experiencing increasing groundwater stress due to intensive agricultural and municipal pumping (ADWR n.d.‐b; Anderson et al. 2007; Aeschbach‐Hertig and Gleeson 2012; Noyes et al. 2025). Natural system changes, such as increased evaporation and declining streamflow, are further compounding these pressures, driven in part by a recent multi‐decadal drought (Williams et al. 2022; Abdelmohsen et al. 2025; Scanlon et al. 2025). Extreme climate events, including droughts, heat waves, and floods, are intensifying, with the extent and duration of extreme events in the US southwest region, resulting in the climate extremes index (CEI) more than doubling between 2010 and 2019 (NOAA n.d.).

Here, we first describe Arizona's groundwater context to lay the foundation for the state as an exemplar of how the ORE framework can be applied. We then describe the existing context of MAR and describe the ORE framework with examples of how recharge efforts could be opportunistically embedded in other land and water management efforts. If successful, these approaches can be applied to other arid and semi‐arid regions in the western United States and globally.

### Arizona Groundwater Context

Early in the 20th century, anticipation of increased demand for water led to efforts to increase supplies by importing water from the Colorado River and a strong commitment to groundwater protection. The landmark 1980 Groundwater Management Act represented a major advancement in water management, forming the Arizona Department of Water Resources (ADWR) to manage statewide water supplies (Jacobs and Holway 2004; Engel et al. 2020). The act also allowed for establishing active management areas (AMAs), putting mandatory conservation requirements in place for all large groundwater uses in AMAs. However, most of Arizona (approximately 75%) is not subject to these regulatory measures, aside from designated irrigation non‐expansion areas (INAs), or the counties that have voluntarily assumed regulatory approaches to ensure water adequacy for new development. In the last few decades, escalating concerns about access to dwindling Colorado River supplies and declining groundwater tables have led to lawsuits as well as numerous commissions and councils appointed to identify solutions to intensifying supply/demand conflicts.

**Figure 1:**
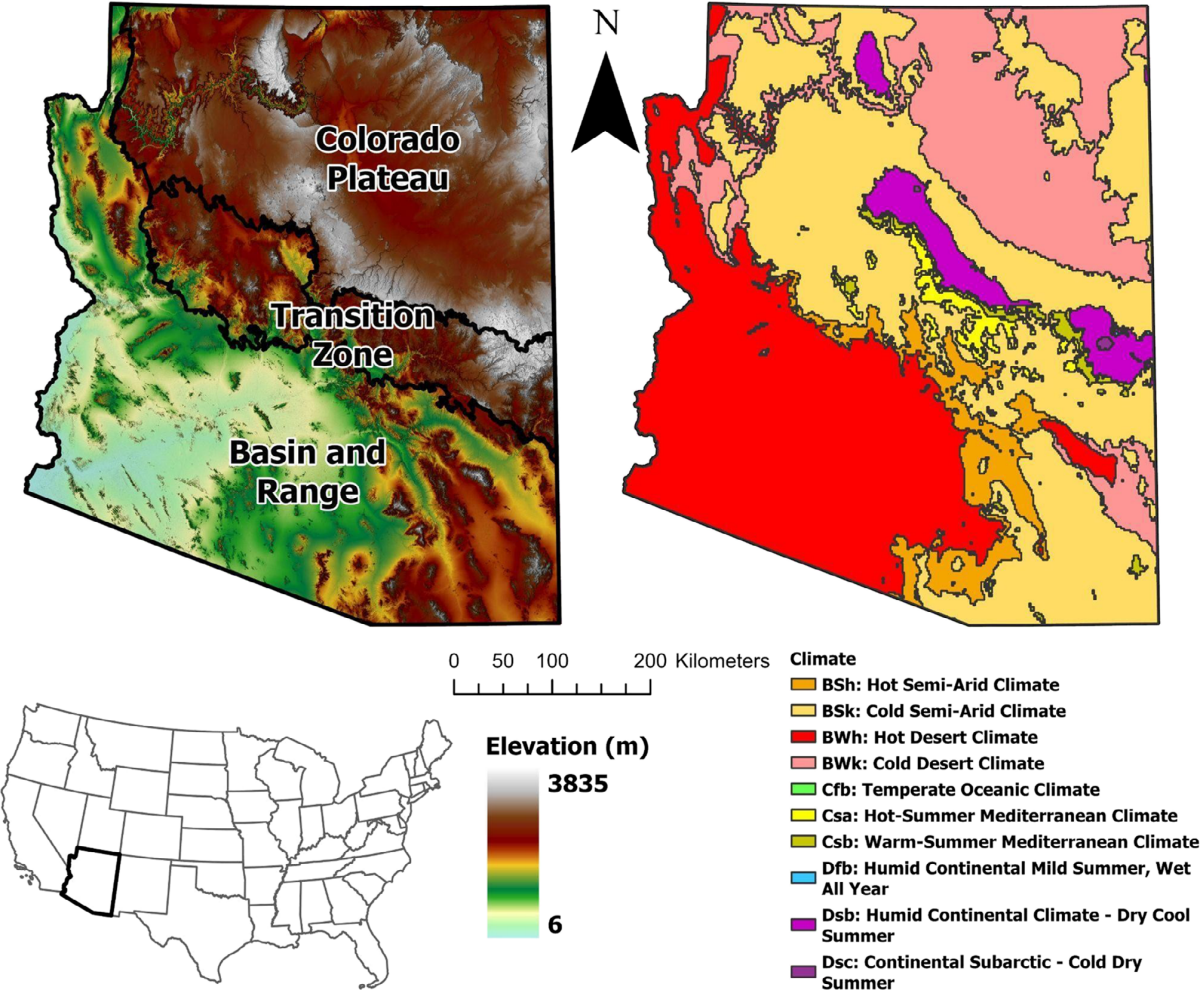
Map of physiographic and climatic diversity in Arizona. Arizona is roughly the size of Italy at around 295,000 km^2^. Arizona contains two major physiographic provinces and a transition zone, with over 3800 m of relief and 10 different climate zones. Climate zones were sourced from ESRI's North American Environmental Atlas—Climate Zones map layer derived from Beck et al. (2018), physiographic provinces were delineated using digital elevation model (DEM), surficial geology, and land cover data.

Arizona is a large and geographically diverse state that ranges from snow‐dominated, high elevation forests on the border of the Colorado Plateau in the north to arid desert valleys in the Basin and Range province in southern Arizona (Figure 1). Natural recharge to Arizona's aquifers is seasonally variable and affected by precipitation magnitude, frequency, intensity, and duration that vary widely across the state (Meixner et al. 2016). In general, increased recharge is observed in winter months, when frontal storm systems bring precipitation and lower temperatures reduce evapotranspiration (ET) relative to the summer monsoon season, the other peak of the state's bimodal precipitation regime (Uhlman et al. 2020).

## Managed Aquifer Recharge (MAR)

MAR is one of many solutions to groundwater shortages. It is a form of anthropogenic water supply enhancement that includes engineered or constructed elements to recharge and/or recover stored water (Dillon et al. 2010). Seven common elements comprise MAR systems (Dillon and Jimenez 2004): water capture, pre‐treatment, recharge, storage, recovery, post‐treatment, and end use. At a minimum, MAR systems include techniques to collect, convey, or intercept water (e.g., diversion dams, canals, weirs) and to enhance infiltration of water (e.g., infiltration basins, injection wells). If the groundwater is to be recovered, wells and pipelines are used to pump and deliver the recovered water after storage. Selection of MAR techniques depends on project goal(s) and must be tailored to specific locations, requiring careful consideration of local context, such as physical site conditions, regulatory constraints, and distance between water sources and recharge zones (Dillon and Jimenez 2004; Dillon et al. 2022). Given these constraints, MAR can be costly. While the implementation of MAR projects is growing at 5% per year, they currently recharge less than 3% of the volume extracted from aquifers worldwide (Dillon et al. 2019). Even optimistic scenarios, assuming cost reductions and widespread adoption, suggest that MAR recharge is limited to 10% of current global groundwater extraction (Dillon et al. 2019).

In Arizona, MAR techniques include spreading basins, injection wells, and permitted discharge to transmissive areas such as streambeds (Arizona Water Banking Authority n.d.). ADWR regulates MAR in Arizona, with considerable permitting requirements intended to protect groundwater aquifers, which are considered a public resource. There are 16 permitted Groundwater Savings Facilities (which exchange surface water for groundwater that would otherwise have been pumped) and 105 Underground Storage Facilities within Arizona (ADWR n.d.‐a), mostly within AMAs. These projects incur significant costs for hydrogeologic surveys prior to permitting, construction, and ongoing operation and maintenance (O&M), and monitoring, with limited financial incentives to build these resource‐intensive projects outside of AMAs. However, MAR projects can incorporate multiple co‐benefits, such as the Sweetwater Recharge Project in Tucson, which recharges reclaimed municipal wastewater, providing water quality and environmental benefits in addition to groundwater recharge (City of Tucson n.d.; Hernandez and Léger 2025).

There is a wide‐range of costs associated with MAR projects, summarized in this section as annualized costs per unit volume over total project costs (capital costs plus operations and maintenance costs). An analysis of 21 projects from five countries (United States, The Netherlands, New Zealand, Australia, and India) found the costs of these projects were between $0.04–$2.67/m^3^ (2016 dollars) to infiltrate water, not including recovery costs (Ross and Hasnain 2018). A subsequent study (Ross 2022) evaluated 21 MAR schemes across 15 countries and found that the wide range of costs (~$0.007 to $1.752/m^3^; 2016 dollars) can be attributed to several factors, including water source (e.g., natural, recycled) and type of infrastructure needed to achieve recharge goals (e.g., basins, injection wells). Infiltration of surface water using infiltration basins or riverbank filtration represented the lower end of costs, while schemes using recycled water or injection wells were found to be much more expensive due to drilling, water treatment, and maintenance costs. Recovery of infiltrated water can also add to the costs (~$0.038 to $5.252/m^3^). An evaluation of costs and benefits of MAR projects in California found similar results, with median cost of $0.33/m^3^ (2015 dollars) (Perrone and Rohde 2016).

## Opportunistic Recharge Enhancement (ORE)

**Figure 2:**
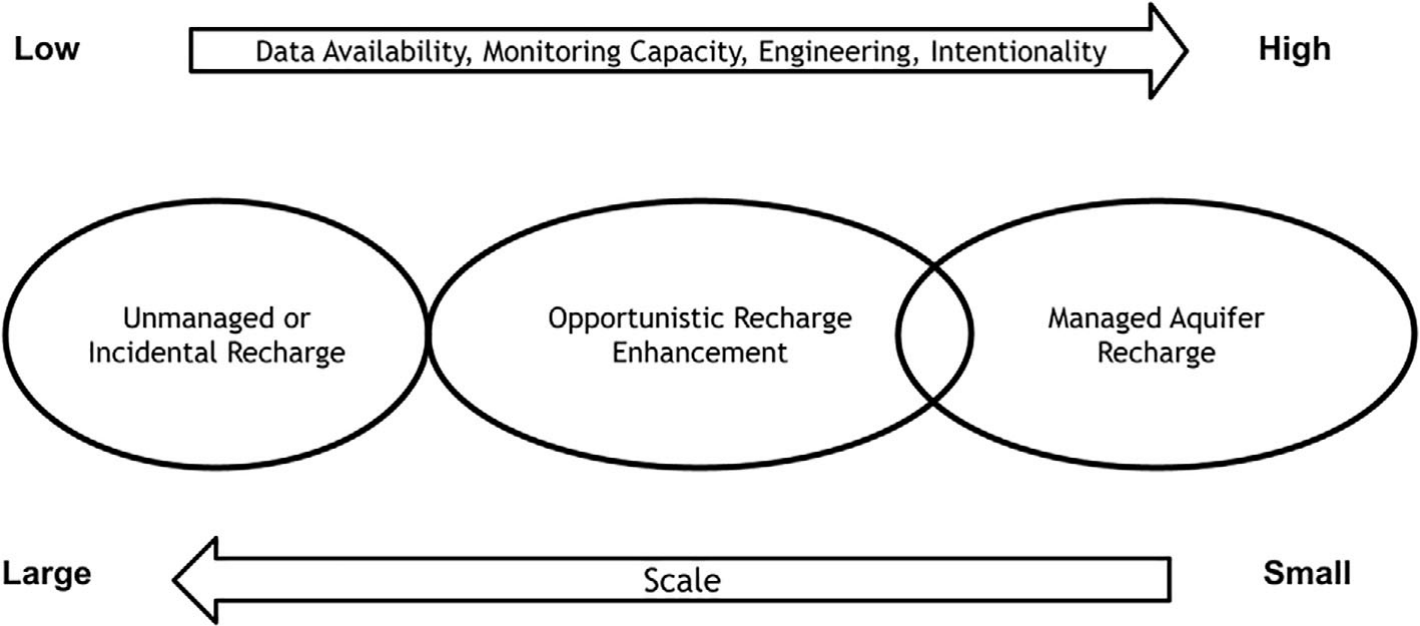
Opportunistic recharge enhancement (ORE) falls between unmanaged or incidental recharge and managed aquifer recharge (MAR) on the recharge spectrum. ORE makes use of natural recharge mechanisms and aims to augment them. ORE is considered supplementary to MAR and may include MAR projects within landscape scale recharge enhancement.

ORE is a concept that can be applied at multiple scales to augment groundwater across landscapes as a co‐benefit of other management efforts (Figure 2). We argue that land and water management activities present opportunities to enhance groundwater recharge as an opportunistic co‐benefit. These approaches can be implemented within the scope of current management activities while limiting the additional costs and bureaucracy of traditional MAR projects. We describe a general framework that incorporates the latest science on land management impacts on recharge, intentionally embedding ORE co‐benefit in decision making.

In contrast to MAR, ORE is primarily focused on increasing recharge by supporting natural recharge processes in the context of other management activities. ORE can be integrated into management actions to achieve several objectives: (1) enhance surface and subsurface water flows, particularly when implemented in headwaters of larger watersheds, (2) directly augment groundwater, where hydrogeology is favorable, and (3) direct water toward groundwater‐dependent ecosystems. A general comparison of the ORE framework and MAR methods is summarized below (Table 1). While there is no “one size fits all” solution for implementing ORE concepts into management strategies, given the wide variation of recharge opportunities across landscapes, management actions, and resource availability, there can be common pathways particularly in areas with large landscapes managed by public agencies.

**Table 1:** Comparison of ORE Framework with Traditional MAR Methods

Category	ORE	MAR (Arizona Context)
Primary objective	Opportunistic recharge as strategic co‐benefit of non‐recharge land management	Deliberate groundwater recharge
Approach	Modify or enhance existing management to increase infiltration and/or reduce evaporative losses	Single purpose infrastructure, including recharge basins or injection wells
Resiliency	Modify human altered ecosystems to enhance water availability	Increase water storage within managed hydrologic systems
Monitoring and accounting	Minimal requirements but may be desirable; natural recharge may be underestimated in water budgets	Closely tracked with permits, modeling, metering, and legal water rights
Flexibility and scalability	Highly scalable; can be designed into or added on to existing projects	Resource intensive, site‐specific; best suited for areas with detailed geologic mapping
Scope and magnitude	Small changes over broad areas, often diffuse recharge	Focused recharge of high volumes in specific spatially constrained locations
Policy/Regulatory fit	Operates outside traditional water rights frameworks, focusing on reducing atmospheric losses or utilizing unallocated water sources	Firmly within regulated groundwater and water rights
Stakeholder engagement	Potential interest from wide range of land‐managers, water providers, NGOs	Primarily engages water providers and water management agencies
Costs	Low to moderate costs; expands new funding opportunities, low operation and maintenance; any additional costs considered marginal and primarily associated with refinement of strategy at location	Moderate to high; capital‐intensive due to design, permitting; costs are primarily associated with project implementation and maintenance
Examples	Alter thinning prescriptions to reduce ET and maintain snowpack, in locations with high infiltration capacity; modify post‐fire flood management; examine recharge potential in endorheic (closed) basins; alter design of flood control or sediment control structures, design features to capture and infiltrate water along roadsides	Spreading basins; aquifer storage and recovery

### Description of ORE Framework

**Figure 3:**
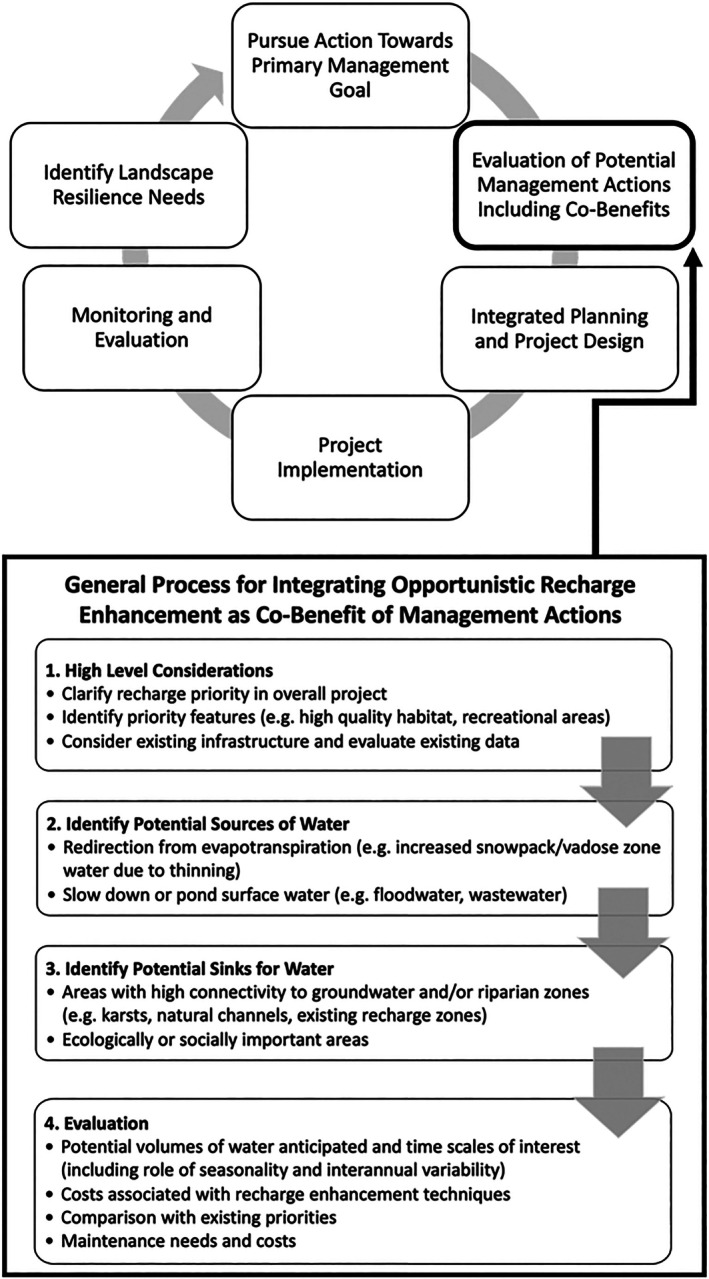
Integrating ORE into a broader adaptive management process, including stages of project prioritization, implementation, and evaluation, is described using the ORE framework throughout the section “Opportunistic Recharge Enhancement (ORE).”

A general framework for integrating ORE in multiple contexts is presented below. While ongoing land and water management activities typically have a primary goal besides recharge, integrating recharge into projects as an intentional co‐benefit may also be feasible and cost‐effective. Successfully integrating ORE would require: (1) clearly defining the objectives and needs of the primary project, (2) evaluating the potential for integrating recharge co‐benefits in the context of the primary project, and (3) a general assessment of the costs and benefits (we are *not* suggesting a traditional “cost–benefit analysis” is required) that includes regulatory, permitting and maintenance implications of a multiple‐benefit project (Figure 3). A key element of ORE planning and implementation is the need for collaboration across management and administrative boundaries, and potentially across agencies to develop alignment toward priority considerations, prospective approaches, and suitable locations for management actions. Forest thinning, controlled burning, irrigated agriculture, dryland farming, stormwater management, and post‐wildfire flood management are all examples of primary activities that, under specific circumstances, can include ORE. Another opportunity worthy of consideration is the incorporation of water capture and recharge into road construction and maintenance projects, especially in sloping areas where overland flow may require culverts or other road protection investments.

Investments in ORE may involve marginal increased costs associated with the refinement of location and design of primary management strategy. This can include the intentional siting of management actions to occur in areas with high infiltration potential and/or connectivity to groundwater. Potential funding mechanisms for ORE projects are expected to largely align with existing funding mechanisms, including internal agency budgets (e.g., County flood control districts), grant opportunities at the state (e.g., Arizona Water Infrastructure Finance Authority, California Department of Water Resources) and the federal level (e.g., US Bureau of Reclamation), and proposition‐defined general obligation bonds (e.g., California; Perrone and Rohde 2016). Calculation of ORE benefits may be complicated due to the variation in source water availability across dry to wet years, in addition to difficulty quantifying the site‐specific costs and benefits in advance. A potential example is the use of green infrastructure funds from the Northern Arizona Forest Fund (NAFF), which is designed to let residents of Arizona invest in the lands and watersheds they depend on. Although NAFF funds are already focused on green infrastructure, they could be intentionally targeted to projects with recharge co‐benefits.

The process of incorporating ORE into management activities can involve multiple pathways, as models of operations vary substantially across management agencies and types of projects. A common starting point is the inclusion of ORE into best management practices (BMPs) with associated metrics aligned with agencies' primary management goal(s); typically, manuals of operation, plans, and standard operating procedures must refer to and align with specific BMPs. The addition of enhanced groundwater recharge within BMPs can support the evaluation and competitiveness of ORE projects. ORE incorporation may be complicated by tradeoffs with other objectives; for example, habitat considerations of forest thinning actions. In these situations, site and regional knowledge, as well as guiding priorities, should be used to consider tradeoffs of ORE incorporation.

### Water Sources

Outside of traditional surface water and municipal/industrial effluent supplies, the potential to increase regional water supplies is highest when water that would otherwise be lost via evaporation, transpiration, or snow sublimation is instead able to infiltrate the soil surface and subsequently recharge groundwater supplies. Opportunities to reduce evaporative losses or harness excess runoff generated due to urbanization or degraded watershed conditions can be prioritized relative to locations where water supply is needed most to sustain human demands or environmental needs. Potential sources of water to increase recharge include accelerated runoff from the impervious surfaces of urbanized areas and rural and wildland watersheds where excessive runoff results in erosion and/or channel incision due to factors such as drought, grazing management practices, post‐fire runoff, or excessive off‐road vehicle use. Sheetflow occurs as unchannelized flow when precipitation intensity exceeds soil infiltration capacity (Hogg 1982; Shaw 2023) and has the potential to provide significant amounts of “new” water for recharge if captured prior to evaporation. In Arizona, sheetflow is considered legally unappropriated surface water until it reaches a defined natural channel. Closed basins with no surface water outflows (endorheic basins) lose water primarily through ET (Wang et al. 2018; Wang 2020) and can contribute to groundwater recharge (Gurdak and Roe 2009; McKenna and Sala 2018). Efforts to reduce ET or increase infiltration in endorheic basins across the state could enhance recharge already occurring (Gurdak and Roe 2009; Salameh et al. 2019) without complicating intra‐ and transbasin water rights.

### Water Sinks

Potential water “sinks” are areas where water can be intercepted and rapidly recharged below the root zone to avoid soil evaporation and transpiration losses. They include areas with high connectivity to groundwater, such as along highly permeable stream channels, areas with shallow groundwater depths, and areas with high secondary (fracture) porosity. Additionally, terrains with karst or pseudo‐karst features such as sinkholes, caves, and lava tubes have high hydraulic conductivity and offer unique opportunities to infiltrate and store water (Ford and Williams 2007; Pulido‐Bosch 2021). Identifying locations of karst topography and high secondary porosity in the landscape could provide significant recharge opportunities in the ORE context if sufficient water is available.

There are multiple mechanisms by which water can be directed and infiltrated into sink areas, including the use of low‐tech structures designed to retain water and sediments, a common range and watershed management practice. These structures come in many forms with many names (e.g., check dams, rock dams, leaky weirs, gabions, earthen berms, Zuni bowls, trincheras) and have been used throughout human history to reduce erosion and capture water and sediment. Collectively, these structures are known as natural infrastructure in dryland streams (NIDS) (Norman et al. 2019). NIDS are often installed for erosion control and may provide multiple recharge co‐benefits (Nichols and Polyakov 2019). NIDSs slow flows and pond water, potentially allowing infiltration into the subsurface. Additionally, the resulting sediment storage increases storage capacity in the subsurface and reduces surface evaporation by storing water within the collected alluvium, (a.k.a. a sand‐dam) (Alley et al. 2022; Petrakis et al. 2024). Further, these structures are designed to allow water to pass through, so they do not result in significant downstream flow reductions and can prolong base flow duration (Norman et al. 2016). In specific circumstances, the infiltrated water may recharge local aquifers, and in most cases, there are demonstrable localized improvements in soil moisture and vegetation health (Norman et al. 2025).

### Analysis and Evaluation

Consideration of ORE opportunities is recommended to occur early in project planning processes and be integrated into project design and selection phases. The early consideration of ORE opportunities includes the identification of potential sources and sinks of water in regions of interest for landscape management interventions. Once potential water sources and sink areas are identified, a preliminary evaluation can be conducted that includes identification of suitable regions to conduct management actions, assessment of co‐benefits of ORE modifications to management strategies, and development of estimates of water volumes to be re‐directed and recharged. These volume estimates will vary between dry and wet years, as well as across seasons, and can also be affected by precipitation intensity and frequency (Wyatt et al. 2015).

In addition to anticipated water volumes, costs, and tradeoffs with other priorities, and maintenance requirements should be considered to guide ORE integration into priority management goals. Costs are likely to be associated with nuances of siting and can be compared to the implementation of management practice without ORE considerations. For example, additional costs may accrue related to water pretreatment, infiltration, or groundwater monitoring in the vicinity of ORE projects. Cost and feasibility are likely to be impacted by factors including land ownership, land use planning requirements, regulatory constraints, and data availability, but these same factors apply to overall project management decisions. Potential increased ecosystem services and other co‐benefits should be considered in addition to more standard valuation processes when assessing potential for ORE, though these benefits can accrue over time and are difficult to assess using traditional valuation methods. The consideration and accounting of recharge benefits in landscape management activities are expected to affect choices in practice by allowing for the evaluation of a broader suite of strategies that are focused on strategic routing and/or retention of water to support enhancement of local groundwater supplies. This can support the identification of opportunities to increase recharge, often at limited costs, that may have otherwise been lost as they went undetected in the pursuit of traditional management goals.

A detailed economic analysis of ORE implementation is beyond the scope of this study. Because of consideration of co‐benefits, there may be a variety of non‐conventional economic approaches that are appropriate for evaluation (Everard 2016; Hérivaux and Grémont 2019). Alternative decision support frameworks, such as multi‐criteria decision analysis (MCDA), can support prioritization and evaluation of projects with multiple goals and co‐benefits through the use of weighted criteria approaches, such as the Analytic Hierarchy Process. The AHP framework can be applied via the development of GIS‐based suitability analyses that allow for the spatial overlay of weighted criteria, including potential areas of high recharge, to support natural resource decision making (Schmoldt et al. 2001; Chakhar and Martel 2004). Willingness‐to‐pay surveys indicate that the value of recharge to long‐term water sustainability could benefit from more detailed groundwater‐based surveys (Mueller et al. 2019; Soder et al. 2022). Stated‐preference or revealed‐preference methods offer a structured approach to quantifying these benefits by eliciting public values for groundwater‐dependent ecosystem services that lack observable market prices. The incorporation of ORE into landscape management activities can support communication and engagement with local communities and stakeholders, providing both environmental and social benefits.

## Examples and Potential Application of ORE in Arizona

In this section, we describe three examples that integrate ORE in management efforts in Arizona.

Beyond a range of applications, these examples also describe the costs and benefits of including ORE at multiple scales (i.e., from across four national forests to local specific drainages).

### ORE Example 1: Forest Thinning/Reductions in Evapotranspiration

One potential application of the ORE framework is through integration with forest management, where the primary ORE strategy involves site selection. In Arizona, major initiatives such as the Four Forest Restoration Initiative (4FRI) aim to treat over a million hectares of forests via mechanical thinning and prescribed burning to mitigate the potential for destructive wildland fires. Forest management is already understood to play a substantial role in watershed management as upstream watershed strategies can benefit many downstream users (Simonit et al. 2015; Robles et al. 2017). The impact of forest thinning has been well documented to reduce vegetation ET (both evaporation/sublimation, from intercepted rainfall/snowfall, and transpiration by the vegetation itself) (Dore et al. 2012; Ha et al. 2015) that will lead to increased surface runoff and/or recharge. In ponderosa pine (*Pinus ponderosa* Douglas ex C. Lawson) forests of northern Arizona, thinning decreased the ratio of ET and precipitation (ET/P) from 1.10 to 0.87 mm/mm during a dry year, and from 0.81 to 0.71 mm/mm during a wet year (Dore et al. 2012). Soil moisture also significantly increased in thinned forests (Belmonte et al. 2022; Sankey and Tatum 2022; Tatum et al. 2025), which in turn provides greater plant‐available water for the remaining trees. Mechanical thinning reduces tree water stress and enhances growth during drought by reducing competition among remaining trees. Given reduced competition and increased moisture availability following thinning, individual ponderosa pine tree‐level transpiration has been documented to increase (Simonin et al. 2007). Canopy moisture in the remaining trees of the thinned forest has also been shown to be significantly greater than non‐thinned forests (Skov et al. 2004; Sankey et al. 2021). Interestingly, thinning also increases understory ET due to larger overstory canopy openings and reduced shading (Simonin et al. 2007). However, total ET in thinned forests is often lower due to reduced tree density compared to non‐thinned forests (Dore et al. 2012; Ha et al. 2015). We argue that when designing and locating forest thinning projects, ORE objectives can be incorporated to maximize project co‐benefits. For example, forest thinning efforts might prioritize areas in karst terrain and areas with high permeability and porosity or above streams, which would enable the reduced ET and associated water savings to recharge groundwater.

Secondly, snow cover duration has been documented to be an important factor influencing runoff and seasonal groundwater recharge (Flint et al. 2004; Earman et al. 2006), and can be managed through thinning forests in a manner that reduces sublimation losses by intentional siting and specific thinning prescriptions that create optimum amounts of tree canopy cover and density. Canopy cover between 25% and 40% appears optimal for net snow accumulation at continental mid‐latitude sites (Veatch et al. 2009), though this optimal value varies with topographic and climate conditions, and may be lower for areas with short‐duration ephemeral snowpacks (Broxton et al. 2020). Shading the snowpack also enhances snow persistence, so a site‐appropriate thinning prescription (including slope/aspect considerations) can promote snowmelt‐driven increases in soil moisture that last later into the year (Belmonte et al. 2022; Dwivedi et al. 2023; Tatum et al. 2025). Unoccupied aerial vehicle (UAV)‐based studies in northern Arizona have shown that 24–35% canopy cover is optimal for creating areas with longer snow persistence (Sankey et al. 2015; Belmonte et al. 2021; Donager et al. 2021) because of the creation of forest gaps where snow is not intercepted (and hence has high accumulation) but still shaded by nearby canopy (and hence has slower ablation). As such, intentionally choosing sites for stand thinning can include shading forest gaps for prolonged snow persistence as an ORE consideration. Water yield enhancement from thinning likely requires at least a 20% reduction in canopy cover (Adams et al. 2012). The optimum prescription will vary for each site, considering appropriate sizes, orientations, and geometry of post‐thinning forest patches that enhance snow accumulation and persistence, while decreasing sublimation, given a site's topographic and climatic conditions. The selected patch geometry needs to be integrated with the goals of at least 20% canopy cover reduction and 30% basal area reduction to improve runoff (Adams et al. 2012; Wyatt et al. 2015). Ecohydrological models in northern Arizona watersheds suggest that reductions in basal area of at least 30% can increase groundwater recharge by up to 15% for up to 7 years if infiltration exceeds threshold levels (Wyatt et al. 2015). In addition, the prescription could focus on areas with high lineament density and potential karst features, and/or consider road grading and flow directions that could be used to enhance groundwater recharge. These intentional decisions could increase water that is available for recharge as well as increase soil moisture later in the summer when drought stress is highest (Belmonte et al. 2021, 2022; Dwivedi et al. 2023).

Thirdly, there is an opportunity to integrate road building and maintenance with recharge considerations. Roads in mountains and crossing hillslopes, in particular, offer enhanced potential for ORE as they frequently have fractured bedrock, receive higher precipitation, and can have shallower vadose zones than adjacent alluvial valleys (Flint et al. 2004; Wilson and Guan 2004). Furthermore, many forest thinning projects occur in such mountainous terrain and often result in building new roads for the forestry operations. Policies that encourage the incorporation of trenching or terracing, grass strips or berms, micro basins, dry wells, and check dams to capture and infiltrate water flowing downslope along roadways as part of future road repair or maintenance in suitable locations have significant potential for enhancing infiltration and recharge (Meles et al. 2024).

Despite the urgent need to ameliorate falling groundwater levels in forested regions across Arizona, aquifer recharge is often a low priority due to myriad other concerns. A choice experiment of metro Phoenix area water users indicated a positive willingness‐to‐pay for watershed restoration but ranked groundwater recharge as the lowest valued attribute (Mueller et al. 2019). More education and outreach by relevant agencies and organizations might improve public recognition of the value of recharge initiatives (Mueller et al. 2019).

### ORE Example 2: Post‐Wildfire Runoff Capture

**Figure 4:**
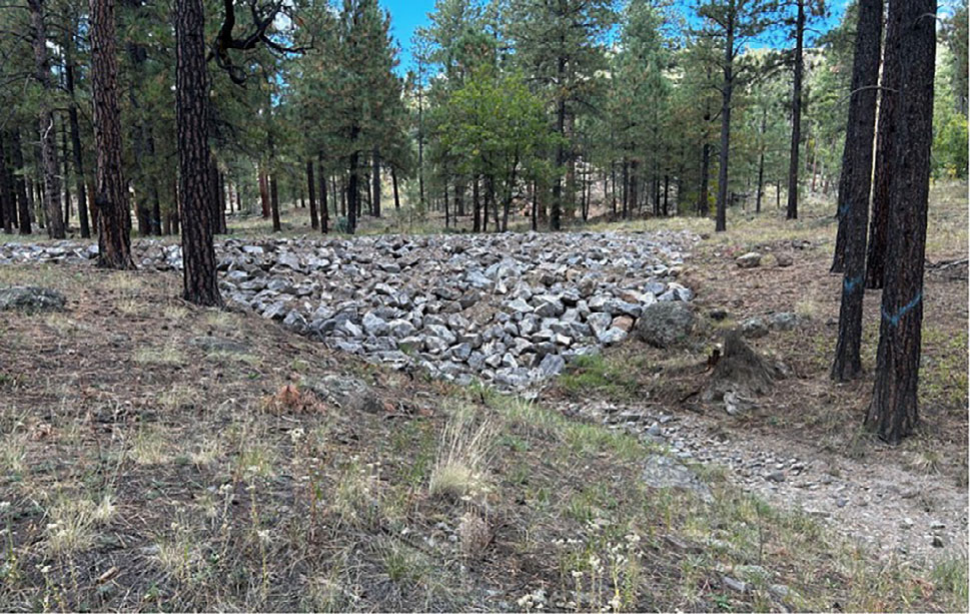
Example of a pond‐and‐plug structure located at the outlet of a highly burned subwatershed of the Rio de Flag. Pond‐and‐plug structures facilitate recharge by capturing post wildfire flood waters and creating a ponded area for water to slowly infiltrate into the substrate. This is one of several structures built in the Rio de Flag Flood Control project.

The ORE framework can be applied to current and future efforts to mitigate post‐wildfire runoff. Wildfires have a significant impact on the hydrologic cycle in arid and semi‐arid regions, impacting slope stability, surface flows, soil conditions, ET, and groundwater recharge. High‐severity burns can create hydrophobic soil layers that significantly reduce infiltration, especially in arid environments with already limited water retention capacity (Beatty and Smith 2013; Van der Sant et al. 2018; Chen et al. 2020). Reduced infiltration capacity often leads to increased runoff and streamflow in burned areas but diminishes local groundwater recharge (Moussoulis et al. 2015; Hallema et al. 2017; Folador et al. 2021). Simultaneously, wildfires reduce vegetation density, which directly lowers transpiration losses and increases water available for streamflow or groundwater recharge (Poon and Kinoshita 2018; Collar et al. 2022). However, impacts to post‐fire ET are not permanent, and ET rates can recover quickly depending on the rate and type of vegetation regeneration (Poon and Kinoshita 2018; Poulos et al. 2021). While reduced ET can enhance streamflow generation by increasing available water, the extent of this effect depends on the proximity of streams to burn scars, as unburned vegetation between burned areas and streams may reabsorb the additional moisture and limit the amount of additional water available for streamflow or recharge (Hallema et al. 2017; Collar et al. 2022). Although soil hydrophobicity generally hinders groundwater recharge within burn scars, wildfire‐induced flooding can temporarily increase recharge downstream or adjacent to burns. These flood waters provide a source of water that can be opportunistically recharged if paired with potential water sinks. In recent years, northern Arizona has experienced much wildfire‐induced flooding in wildland‐urban interface areas (Sankey et al. 2024), and such events are projected to increase. Concerns about post‐wildfire flood impacts have led the city of Flagstaff in northern Arizona to build many detention basins to reduce post‐wildfire flood impacts (Figure 4). These basins have been developed primarily to manage debris and flood flows but could easily be modified to enhance recharge in selected locations. Adding this management objective to the overall approach could result in increased local water supply without significant additional costs.

A potential application site for these techniques is the Rio de Flag, a steep forested stream that drains a portion of the San Francisco Peaks near Flagstaff before draining into the Little Colorado River. Recent wildfires on the San Francisco Peaks in 2010, 2019, 2021, and 2022 have damaged the forests' ability to mediate storm runoff, causing flooding and high flood risk for the City of Flagstaff (Sankey et al. 2024). The Rio de Flag Flood Control project is a $122 million dollar effort with the goal of reducing flood and sediment risk to the City of Flagstaff. While the main goal is to reduce flood‐associated damages to the downstream community, many of the project components may allow recharge to be enhanced opportunistically, with the consideration of basin locations and subsurface hydrogeology. For example, the project created retention ponds to collect sediment and flood water before it enters the Rio de Flag. These retention ponds, placed at the outlet of a highly burned subwatershed, hold over 50 acre‐feet of water at full capacity. By retaining water for long periods, the ponds allow for longer residence time leading to more opportunity for water to infiltrate to the subsurface. Large precipitation events such as the 2023 winter snows and associated runoff filled the ponds to capacity and retained water for several weeks, highlighting the importance of seasonality when considering ORE evaluation. The project also included constructed alluvial fans and pond‐and‐plug structures (Figure 4) that enhance sheet flow and facilitate infiltration in areas with high permeability before entering the stream channel. In addition to the large structures at the outlet of the subwatershed, the project also included the construction of many NIDS within the channel. Although these structures may not amount to the same volumetric contributions to recharge as the larger retention basins, NIDS still provide environmental benefits such as increased soil moisture, infiltration, and snowmelt ponding.

By considering ORE in post wildfire flood control site selection, water can be retained or re‐routed to areas with high permeability or connection to groundwater, thereby reducing flood risk and enhancing groundwater recharge simultaneously. In areas like northern Arizona, which overlay karst and volcanic lithology, flood waters may be re‐routed to natural sink holes or highly fractured areas to ensure infiltration to the subsurface. There may also be highly suitable locations in or near developed areas for drywells to be installed alongside retention ponds to quickly route nuisance floodwater into the subsurface and reduce losses to ET. Other considerations for post wildfire infrastructure include lining retention ponds with highly permeable material to enhance rapid infiltration and reduce ET or placing ponds in ephemeral channels with high transmission losses. However, water quality is an important consideration for post wildfire ORE practices and may impact design considerations. Post‐wildfire flooding provides a water source, and implementing ORE could help mitigate flood risks as well as connect the additional source of water to potential sinks. It could be integrated within the planning and decision process in preparation for future fire/flood risks relatively easily. In many communities, stormwater management is handled separately from water resources. The ORE framework could be a bridge between local agencies and departments to gain added benefits from investments in resource management.

### ORE Example 3: Urban Stormwater Management

ORE has potential applications in urban stormwater management, addressing excess runoff generated when impervious surfaces replace natural pervious areas. Stormwater management objectives have historically focused on reducing risk to life and property (Fry and Maxwell 2017) by diverting water to large detention basins using hardened channels in the urban landscape, with recharge of aquifers rarely mentioned as an objective. Cities in arid regions are increasingly viewing stormwater as a source of episodic water that can be used to directly increase groundwater recharge or to reduce pressures upon potable water supplies (Crosson et al. 2018; Wilfong and Pavao‐Zuckerman 2020; Gerlak et al. 2021; Korgaonkar et al. 2021; Thomson 2021; Tucson Water 2023; Aloui et al. 2024; Crosson et al. 2024) while providing environmental co‐benefits such as reducing heat and air pollution mitigation and improving health outcomes (Coutts and Hahn 2015; Norton et al. 2015; Bottalico et al. 2016; Ortolani and Vitale 2016; Abhijith et al. 2017; Douglas et al. 2017; Suppakittpaisarn et al. 2017). Stormwater management features such as drywells (Graf 2015) have the potential to capture significant volumes of stormwater while minimizing evaporative losses, depending on their placement and contributing catchment areas. One study estimated upwards of 100,000 acre‐feet per year of stormwater capture through drywells and detention/retention basins in the Phoenix AMA (Su et al. 2025). This demonstrated efficacy in the Phoenix area highlights the opportunity for other Arizona municipalities to leverage existing and new stormwater infrastructure for regional groundwater replenishment efforts.

In the Upper San Pedro River Basin, urban runoff from the City of Sierra Vista drains into natural ephemeral channels, the largest of which is Coyote Wash. However, flood peaks in Coyote Wash have significantly increased due to runoff from impervious surfaces in the urban landscape, resulting in exacerbated flooding, erosion, and sedimentation. Even though the coarse‐grained beds of these ephemeral channels are known for their important role in regional aquifer recharge, the higher magnitude, short duration storm events did not have adequate time for infiltration and recharge to occur in the beds of streams draining urbanized areas.

A surface water diversion dam has been constructed to send flood flows from Coyote Wash to an adjacent abandoned gravel pit, where it is slowly metered back into the channel, allowing for more time for infiltration and recharge to occur within the channel. This nature‐based approach is more effective than simply capturing the urban runoff in a traditional flood control detention basin outside the floodplain, where evaporative losses would have been large, deep impermeable clay layers would prevent recharge from occurring, and both construction and maintenance costs would be relatively high. As a result of the project, flood events will more closely resemble the pre‐development flood flow regime, providing benefit to the high‐quality riparian ecosystem in the San Pedro Riparian National Conservation Area (SPRNCA) downstream. A similar approach is being taken along Horseshoe Draw (CCRN n.d.), an ephemeral channel in the same watershed that conveys accelerated runoff from rangelands upstream in Mexico into a constructed sediment control basin. The basin also slows flows enough to allow for additional recharge in the bed of the natural ephemeral channel downstream than would have occurred without the basin.

In these examples, recharge co‐benefits were explicitly considered as part of both flood control and sediment control early on in project planning and design. This was made possible through the collaboration of local, state, and federal agencies, along with private landowners and donors. Together these partners were able to secure a broader array of funding sources to launch the projects and reduce the total number of projects needed to meet their various objectives, as well as long‐term operation and maintenance costs. These projects, along with six other groundwater management sites, are intended to enhance near‐stream groundwater levels to support riparian health along 25 miles of the SPRNCA and are collaboratively managed by the interagency efforts of the Cochise Conservation and Recharge Network (CCRN).

The economic benefit of this network of projects is connected to the maintenance of riparian health within the SPRNCA for federally listed endangered riparian obligate species and their critical habitat, such as the yellow‐billed cuckoo. Together these projects help to mitigate groundwater pumping in the region that could impact the riparian area, such that other federal agency partners in the basin, including the Department of Defense at Fort Huachuca, can continue their military mission which provides $2 billion annually to the economy of southern Arizona and the State.

## Conclusions

Groundwater availability is declining around the world, yet there is a range of strategies that is yet to be fully explored and deployed at scale. Recharge efforts are typically conducted using a MAR framework that relies on engineered solutions to augment groundwater. We present additional opportunities through opportunistic recharge enhancement or ORE, an approach that leverages aspects of both managed and natural recharge processes. The ORE framework explicitly considers the potential recharge benefits of modifying existing management actions to incorporate recharge enhancement. There is no “one‐size fits all” pathway for ORE, as strategies can vary widely across landscapes and agency management priorities. ORE generally requires collaboration across management boundaries and agencies to agree upon priority actions, locations, and processes to accomplish intended goals. However, several ORE options are within the jurisdiction of one agency, such as the U.S. Forest Service. A key element of ORE planning and implementation is the need for broadening the range of possible benefits of individual projects; collaboration across management boundaries and agencies to develop alignment toward priority considerations may be required to identify suitable locations for ORE implementation.

ORE considers the complex interactions between vegetation, water fluxes, and subsurface geological features to identify opportunities that can be integrated into ongoing land and water management efforts in urban, rural, and natural landscapes at multiple scales, with reduced regulatory requirements. Examples of existing management actions that would align with the ORE framework include road maintenance, post‐fire flood mitigation, and the adoption of green stormwater infrastructure. Often, these projects are implemented for reasons unrelated or tangential to recharge, such as erosion control, infrastructure protection, habitat restoration, flood risk reduction, and wildfire risk reduction. From this perspective, ORE complements MAR by expanding the palette of recharge‐enhancing strategies beyond capital‐intensive projects to include multi‐benefit interventions that are diffuse and scalable.

Costs associated with ORE are expected to be marginal compared to MAR projects, as ORE is not a single‐purpose recharge intervention. MAR project costs are typically related to permitting, construction, monitoring, geologic site characterization, and water recovery operations. ORE, however, emphasizes modifying proposed management actions to enhance recharge; in general, ORE involves marginal costs associated with the refinement of location and design of the primary management strategy.

We believe this framework could result in significant increases in the volume of water recharged within arid and semi‐arid landscapes at relatively low cost, with both social and environmental benefits. One near term step to implementation of ORE is the inclusion of this concept as a management goal within agency BMPs, comprehensive plans and regional management plans. This simple addition could lead to a rapid increase in projects that incorporate ORE by making projects more explicit in their alignment with agency management priorities. ORE incorporation may be complicated by tradeoffs with other objectives, for example habitat considerations of forest thinning actions. In these situations, site and regional knowledge, as well as guiding priorities, should be used to consider tradeoffs of ORE incorporation.

There are several near‐term actions and research priorities that can support implementation of ORE concepts, including identification of existing land use planning processes that require regular updates (e.g., county comprehensive plans, forest management plants) to identify where there may be mechanisms to insert these ideas. Additionally, wider availability of decision support tools, including but not limited to more documentation of costs and benefits, development of enhanced monitoring to support regional groundwater models, etc., that can support evaluation and decision‐making processes related to ORE. An inventory of existing relevant decision support tools and case studies would be an initial step for regions and agencies interested in pursuing ORE implementation. Expanded environmental monitoring and paired watershed studies can provide valuable data to support evaluation of changes in groundwater recharge due to landscape management actions. Finally, we recommend that outcomes and lessons learned from ORE implementation be documented and shared to assess the potential these various approaches can offer land and water managers over time.

## Data Availability

Data sharing not applicable to this article as no datasets were generated or analysed during the current study.
